# Epigenetic changes associated with disease progression in a mouse model of childhood allergic asthma

**DOI:** 10.1242/dmm.011247

**Published:** 2013-04-23

**Authors:** Adam Collison, Jessica S. Siegle, Nicole G. Hansbro, Chau-To Kwok, Cristan Herbert, Joerg Mattes, Megan Hitchins, Paul S. Foster, Rakesh K. Kumar

**Affiliations:** 1 Centre for Asthma and Respiratory Disease, University of Newcastle and Hunter Medical Research Institute, Newcastle, NSW 2308, Australia; 2 Inflammation and Infection Research Centre, School of Medical Sciences, University of New South Wales, Sydney, NSW 2052, Australia; 3 Medical Epigenetics Laboratory, Lowy Cancer Research Centre, University of New South Wales, Sydney, NSW 2052, Australia

## Abstract

Development of asthma in childhood is linked to viral infections of the lower respiratory tract in early life, with subsequent chronic exposure to allergens. Progression to persistent asthma is associated with a Th2-biased immunological response and structural remodelling of the airways. The underlying mechanisms are unclear, but could involve epigenetic changes. To investigate this, we employed a recently developed mouse model in which self-limited neonatal infection with a pneumovirus, followed by sensitisation to ovalbumin via the respiratory tract and low-level chronic challenge with aerosolised antigen, leads to development of an asthmatic phenotype. We assessed expression of microRNA by cells in the proximal airways, comparing changes over the period of disease progression, and used target prediction databases to identify genes likely to be up- or downregulated as a consequence of altered regulation of microRNA. In parallel, we assessed DNA methylation in pulmonary CD4^+^ T cells. We found that a limited number of microRNAs exhibited marked up- or downregulation following early-life infection and sensitisation, for many of which the levels of expression were further changed following chronic challenge with the sensitizing antigen. Targets of these microRNAs included genes involved in immune or inflammatory responses (e.g. *Gata3*, *Kitl*) and in tissue remodelling (e.g. *Igf1*, *Tgfbr1*), as well as genes for various transcription factors and signalling proteins. In pulmonary CD4^+^ T cells, there was significant demethylation at promoter sites for interleukin-4 and interferon-γ, the latter increasing following chronic challenge. We conclude that, in this model, progression to an asthmatic phenotype is linked to epigenetic regulation of genes associated with inflammation and structural remodelling, and with T-cell commitment to a Th2 immunological response. Epigenetic changes associated with this pattern of gene activation might play a role in the development of childhood asthma.

## INTRODUCTION

Asthma in childhood is strongly linked to atopy and a Th2-biased immunological response to chronic allergen exposure (Sly et al., 2008; Holt and Sly, 2012). Although genetics clearly play a role, environmental factors seem to be important in the development of the asthmatic phenotype. Birth cohort studies suggest that there is a greatly increased risk of development of clinical features of asthma in children who suffer wheezing lower respiratory tract infections in early life, notably with rhinovirus and respiratory syncytial virus (RSV), on a background of sensitisation to aeroallergens (Kusel et al., 2007; Jackson et al., 2012; Kusel et al., 2012). The underlying mechanisms involved have not yet been defined, although there is much interest in the cross-talk between innate host defence responses (including the responses of airway epithelial cells) and the adaptive immune response (Sabroe et al., 2007; Holt and Strickland, 2010; Kumar et al., 2011).

Birth cohort studies have also established that, whereas wheezing is common in early childhood, this is usually transient and resolves spontaneously (Taussig et al., 2003). Progression to persistent asthma is likely to be related to relatively stable changes in the immunological response and/or to structural changes in the airway wall referred to as airway remodelling, which might in turn be a consequence of epigenetic changes, possibly driven by environmental factors (Kumar et al., 2009; Martino and Prescott, 2011). However, it is difficult to investigate such changes and to assess the altered immunological response in the airway wall in children with asthma.

We have recently described a model of the interaction between early-life respiratory viral infection and allergen exposure in the development of an asthmatic phenotype in mice (Siegle et al., 2010). In this model, animals are neonatally infected with pneumonia virus of mice (PVM), which belongs to the same family and genus as RSV but is a natural rodent pathogen (unlike human RSV, which exhibits limited replication in mice) and can thus model the full spectrum of pathological changes of human RSV disease in early life (Domachowske et al., 2004; Rosenberg and Domachowske, 2008). A low-inoculum infection allows spontaneous recovery, following which sensitisation to ovalbumin (OVA) via the respiratory tract and long-term aerosol challenge are used to simulate human allergen exposure, using mass concentrations of aerosolised antigen at least 10- to 100-fold lower than in commonly used short-term experimental systems. We showed that, although some features of asthma, such as hyper-responsiveness to a cholinergic stimulus or epithelial remodelling, developed in response to either viral infection or allergen challenge, a complete asthmatic phenotype was evident only in animals that had recovered from early-life infection with PVM and then received chronic allergen challenge. Furthermore, development of allergic inflammation with recruitment of eosinophils was dependent on the accumulation and activation of pulmonary T cells, with induction of a Th2-biased immunological response (Siegle et al., 2010).

**TRANSLATIONAL IMPACT**

**Clinical issue**

Asthma is a common chronic inflammatory disease of the airways that is thought to be caused by a combination of genetic and environmental factors. Development of asthma during childhood is linked to viral infections of the lower respiratory tract, with subsequent chronic exposure to allergens. The mechanisms underlying progression to persistent asthma, characterised by the development of a relatively stable Th2-biased immunological response and structural remodelling of the airways, remain unclear. It has been postulated that epigenetic changes could be involved in shaping these events; however, thus far it has proven difficult to assess these changes in child asthmatics and early experimental models.

**Results**

Here, the authors investigated the epigenetic changes associated with disease progression using a mouse model that they recently established. In this model, a complete asthmatic phenotype is induced by early-life pneumovirus infection, respiratory sensitisation and low-level chronic antigenic challenge. The group assessed the expression of microRNAs in the airway wall of affected mice, as well as DNA methylation in pulmonary CD4^+^ T cells. They identified a number of microRNA target genes that were significantly upregulated or downregulated over the period of chronic challenge with sensitising antigen; notably, genes that are involved in immune or inflammatory responses and in tissue remodelling, in addition to genes encoding various transcription factors and signalling proteins. Furthermore, their analysis revealed significant demethylation at promoter sites for interleukin-4 and interferon-γ in pulmonary CD4^+^ T cells.

**Implications and future directions**

These results provide new insights into the molecular mechanisms involved in the development of childhood asthma. Collectively, the data show that upregulation of genes associated with inflammation and structural remodelling, driven by epigenetic changes, together with regulation of T-cell commitment to a Th2-biased immunological response, could constitute a biologically relevant pathway associated with progression to an asthmatic phenotype. In addition to highlighting target genes that warrant further analysis, the work validates the utility of the mouse model for the investigation of childhood asthma pathogenesis.

Our model thus has particular advantages for examining the evolution of inflammatory, immunological and structural changes during the induction phase of childhood asthma. In the present study, we have employed this model to investigate the epigenetic changes associated with early-life viral infection and chronic allergen challenge. We assessed altered expression of microRNAs (miRNAs) in the airway wall, and the temporal relationship between disease progression and predicted expression of genes that would be up- or downregulated as a consequence of altered epigenetic regulation. In addition, we assessed epigenetic changes associated with the phenotypic commitment of CD4^+^ T cells, including DNA methylation of genes linked to the emergence of stable Th2 or Th1 differentiation. Such studies have not previously been undertaken. Our findings suggest that the progression of childhood asthma is linked to altered regulation of inflammatory pathways, a distinctive pattern of altered gene expression associated with structural remodelling in the airway wall, and increased expression of both interleukin 4 (IL-4; encoded by *Il4*) and interferon-γ (IFN-γ; encoded by *Ifng*) by CD4^+^ T cells.

## RESULTS

### Altered miRNA expression in the airway wall

To evaluate changes in the relative expression of various miRNAs in blunt-dissected airway wall tissue from sensitised and challenged animals, we analysed microarray data from four samples per group and identified miRNAs that exhibited large mean fold changes at days 49 and 77 compared with naive mice, or large mean fold changes between days 49 and 77. At day 49 of life, following recovery from neonatal PVM infection and sensitisation via the respiratory tract but prior to any inhalational challenge with OVA, there was marked (≥4.5-fold) upregulation of a limited subset of six miRNAs as assessed by analysis of microarray data (Table 1). At day 77 of life, following 4 weeks of low-level chronic inhalational challenge and a single moderate-level challenge with aerosolised OVA, four of these miRNAs were still markedly upregulated, and an additional 11 miRNAs were upregulated. Similarly, at day 49, a limited subset of six miRNAs exhibited marked (≥4.5-fold) downregulation and, at day 77, an additional 18 miRNAs were strikingly downregulated (Table 1).

**Table 1.**
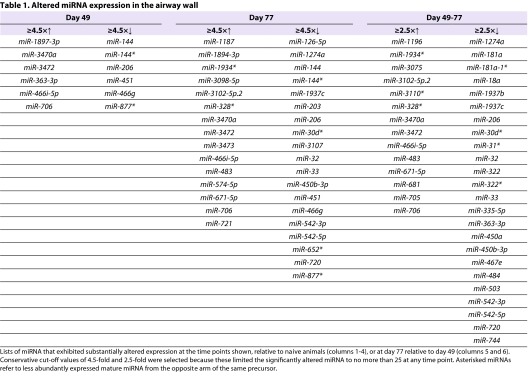
Altered miRNA expression in the airway wall

**Fig. 1.**
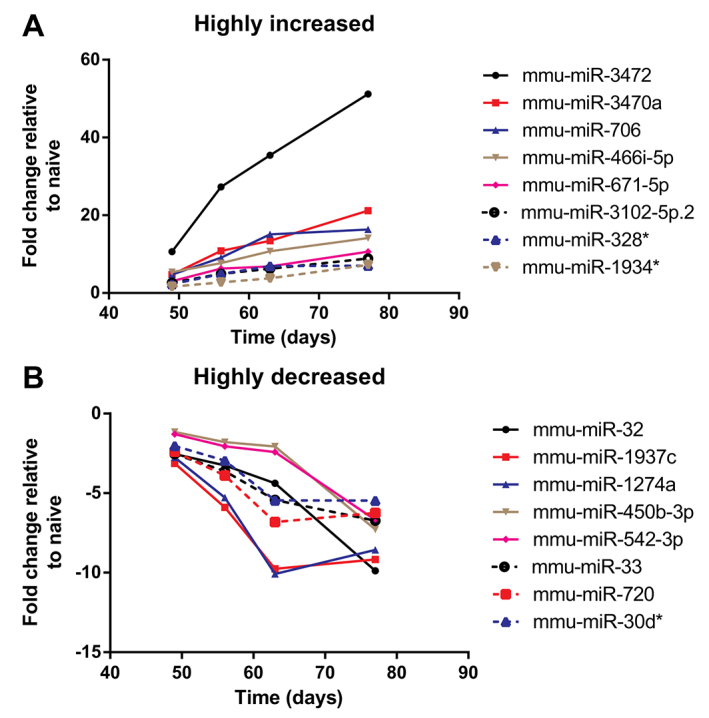
**Selected examples of highly regulated miRNAs.** Mean relative expression of miRNAs that exhibited (A) the greatest increases or (B) the greatest decreases between the beginning and the end of chronic challenge.

For several miRNAs, levels of expression progressively changed during the period of chronic inhalational challenge, with values at day 77 differing by ≥2.5-fold both from values at day 49 and from naive animals (Table 1). Changes over time for a selection of miRNAs that exhibited the highest levels of upregulation by day 77 are shown in Fig. 1A, and a selection of those that exhibited the greatest downregulation are shown in Fig. 1B.

**Fig. 2.**
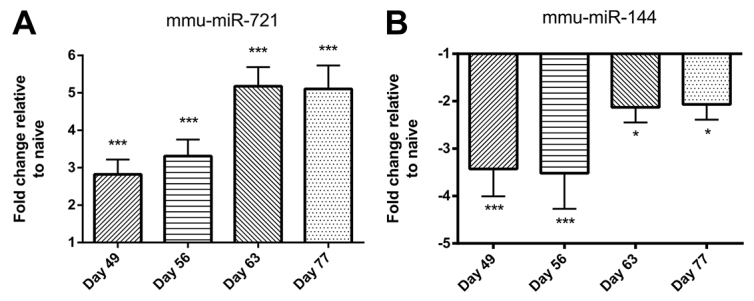
**qRT-PCR confirmation of up- or downregulation of selected miRNAs in airway wall tissue of animals over the period of chronic challenge.** (A) Increased expression of *mmu-miR-721.* (B) Decreased expression of *mmu-miR-144*. Data are mean ± s.e.m. (*n*=6 samples per group). Significant differences compared with naive controls are shown as *P<0.05 and ***P<0.001.

The changes in level of expression of selected miRNAs were confirmed by quantitative reverse-transcription PCR (qRT-PCR). Increased expression of *mmu-miR-721* is shown in Fig. 2A and decreased expression of *mmu-miR-144* in Fig. 2B.

### Targets of markedly up- or downregulated miRNAs

To understand the significance of the changes in expression of miRNA in the airway wall, we used GeneSpring XI software to generate lists of genes that were potential targets of highly upregulated or downregulated miRNAs, limiting these to conserved miRNAs and to targets ranked above the 95th percentile via the TargetScan database. These lists (100–600 genes per set, six sets corresponding to the columns in Table 1) were then carefully examined to identify genes that might be relevant to the immunological or inflammatory response, to tissue remodelling, or to the signalling pathways relevant to these processes (Table 2).

**Table 2.**
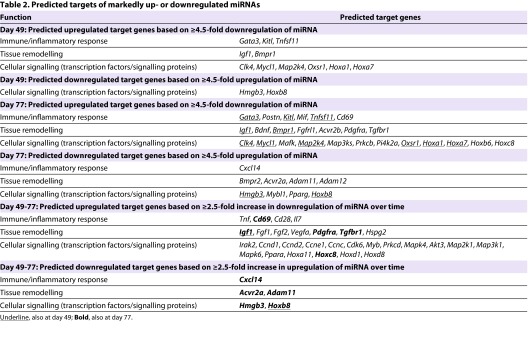
Predicted targets of markedly up- or downregulated miRNAs

At day 49, notable genes predicted to be upregulated included *Gata3*, the transcription factor associated with Th2 differentiation of CD4^+^ T cells, *Kitl* or stem cell factor, and *Igf1* or insulin-like growth factor-1, which is associated with airway remodelling. In parallel there was predicted upregulation of *Bmpr1*, the type 1 receptor for bone morphogenetic proteins, which belong to the transforming growth factor-β family. There was also predicted upregulation of the homeobox genes *Hoxa1* and *Hoxa7*, but downregulation of *Hoxb8*.

At day 77, *Gata3* was still predicted to be upregulated, as was *Postn*, which codes for periostin, induced in the setting of allergic inflammation. *Kitl* continued to be upregulated, and additional inflammation-relevant genes that were predicted to exhibit increased expression included *Mif*, involved in macrophage activation, and *Cd69*. Also noteworthy was that *Igf1* continued to be upregulated, as did *Bmpr1*, but *Bmpr2* was predicted to be downregulated. Other receptors for growth factors that were predicted to be upregulated included *Pdgfra* [encoding the receptor for platelet-derived growth factor (PDGF)], *Tgfbr1* [for transforming growth factor-β (TGF-β)] and *Acvr2b* (for activin), but *Acvr2a* was downregulated. Interestingly, *Hoxa1* and *Hoxa7* continued to be predicted as upregulated, together with other homeobox proteins, whereas *Hoxb8* remained downregulated.

At both day 49 and 77, numerous signalling pathway proteins and cell cycle regulatory proteins seemed to be regulated by miRNA. These included a variety of upregulated kinases associated with inflammation (Table 2).

Examination of predicted targets for those miRNAs that exhibited the greatest changes between day 49 and 77 revealed several genes common to the earlier lists: for example, genes encoding remodelling-associated growth factors and receptors (such as *Igf1*, *Pdgfra* and *Tgfbr1*) were upregulated. In addition, members of the fibroblast growth factor family such as *Fgf1* and *Fgf7* (also known as keratinocyte growth factor) were predicted to be upregulated. Among inflammation-associated mediators, the predicted upregulation of *Tnf* [encoding the key cytokine tumour necrosis factor-α (TNFα)] and the T-cell costimulatory molecule *Cd28* were both of particular interest.

**Fig. 3.**
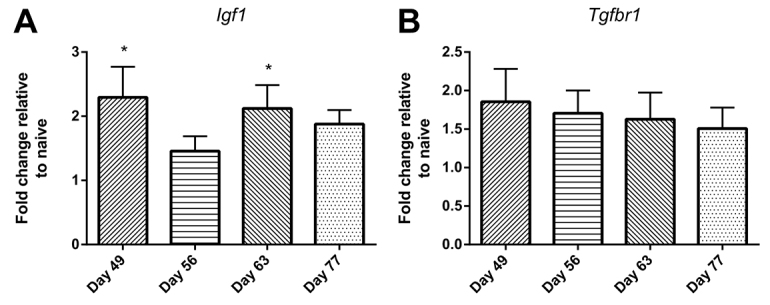
**qRT-PCR assessment of the upregulation of predicted mRNAs in airway wall tissue of animals over the period of chronic challenge.** (A) Increased expression of *Igf1.* (B) Increased expression of *Tgfbr1*. Data are mean ± s.e.m. (*n*=4–6 samples per group). Significant differences compared with naive controls are shown as *P<0.05.

Confirmation of increased levels of expression of mRNAs that were predicted targets of downregulated miRNAs was complicated by the development of inflammation in the airway walls, which increases the number of cells in the tissue and thus the denominator relative to which mRNA expression is normalised. Nevertheless, using qRT-PCR we demonstrated that relative expression of the remodelling-associated gene *Igf1* was significantly elevated at days 49 and 63 (Fig. 3A). There was also a modest increase in expression of the *Tgfbr1* gene, although this was not statistically significant (Fig. 3B).

### Altered DNA methylation in the *Il-4* and *Ifng* promoter regions in CD4^+^ T cells

**Fig. 4.**
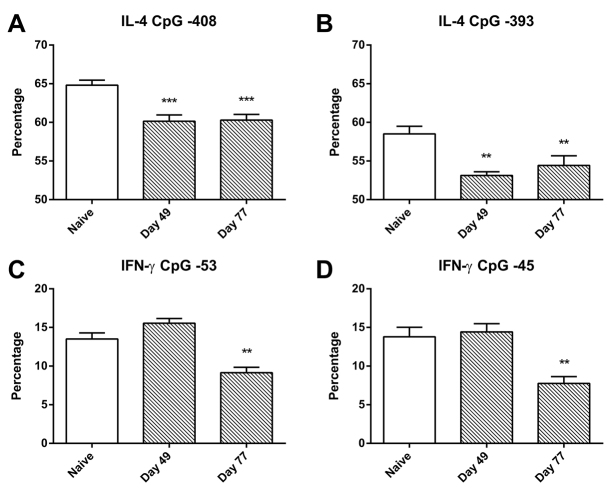
**Percentage methylation of CpG islands in *Il4* and *Ifng* promoter regions in CD4^+^ T cells.** (A) Decreased methylation of *Il4* CpG at −408. (B) Decreased methylation of *Il4* CpG at −393. (C) Decreased methylation of *Ifng* CpG at −53. (D) Decreased methylation of *Ifng* CpG at −45. Data are mean ± s.e.m. (*n*=6 samples per group). Significant differences compared with naive controls are shown as **P<0.01 and ***P<0.001.

To assess the epigenetic changes in pulmonary CD4^+^ T cells associated with the induction of a Th2-biased immunological response, we examined the methylation levels at particular CpG sites upstream of the transcription initiation sites of the *Il-4* and *Ifng* genes in DNA from purified CD4^+^ T cells from lung-draining lymph nodes of individual animals. We found that, at the −408 and −393 sites in the promoter region of *Il-4*, there was evidence of significant demethylation at both day 49 and day 77, although there was no further demethylation over this time period (Fig. 4A,B). No such demethylation was evident at the −16 CpG site in the *Il-4* promoter region (not shown).

At day 49, the percentage of methylated DNA in the *Ifng* promoter region was essentially identical to that in naive animals, whereas, at day 77 after long-term inhalational challenge, the percentage of methylated DNA at two of the CpG sites in the *Ifng* promoter region (−53, −45) was approximately halved (Fig. 4C,D). No such change was seen at the third CpG site (−34) (not shown).

## DISCUSSION

Development of asthma in children is predisposed to by early-life lower respiratory viral infections of sufficient severity to cause wheezing (Sly et al., 2010), and seems to be dependent on sensitisation (Jackson et al., 2012) as well as subsequent chronic exposure to environmental allergens (Holt and Sly, 2009). The biological events associated with such ongoing allergen challenge remain largely undefined. In our animal model, we have previously demonstrated that development of an asthmatic phenotype (including airway inflammation, remodelling and hyper-responsiveness to a cholinergic bronchoconstrictor stimulus) is dependent on the interaction between early-life viral infection, sensitisation via the respiratory tract and chronic exposure to aerosolised antigen (Siegle et al., 2010). This model thus simulates key features of the onset of childhood asthma and facilitates investigation of its pathogenesis. In the present study, we sought to define epigenetic changes and molecular mechanisms associated with the evolution of the disease process.

For this purpose, we examined altered DNA methylation that was associated with the expression of specific cytokines by pulmonary CD4^+^ T cells, as well as examining changes in the expression of miRNAs by cells in the airway wall, comparing responses before and after chronic inhalational challenge with antigen. For assessment of changes in miRNA, we adopted a conservative approach, focusing on miRNAs that exhibited relatively large fold changes and that were conserved across species. Having identified miRNAs that were differentially expressed, we inferred altered regulation of biologically relevant mediators using target prediction databases. This can be a powerful way of identifying unknown pathways of relevance to the pathogenesis of disease, as recently demonstrated in a comparative study of the expression of miRNA by airway epithelial cells from asthmatic and non-asthmatic individuals (Jardim et al., 2012).

Our previously published studies had shown that development of an augmented Th2-biased immunological response required both recovery from early-life infection with PVM and chronic challenge with OVA. This was associated with enhanced expression by CD4^+^ T cells of the prototypic Th2 cytokines IL-4 and IL-13, as well as of the transcription factor GATA-3 (Siegle et al., 2010), which is crucially associated with Th2 differentiation and the development of allergic inflammation (Sel et al., 2008). The present results indicate that a bias towards development of a Th2 response is present even prior to the commencement of inhalational challenge, because downregulation of miRNA in the airway wall predicted upregulation of GATA-3 as early as day 49 of life. Concurrently, there was demethylation of the *Il-4* locus in pulmonary CD4^+^ T cells, a finding of considerable interest given that such epigenetic changes are now recognised as the basis for development of a stably altered immunological response (van Panhuys et al., 2008; Zhu and Paul, 2008). In addition, there was predicted early upregulation of Kit ligand or stem cell factor, which could be related to IL-13-stimulated production of this cytokine by airway epithelial cells in a Th2 environment, and might drive the accumulation of mast cells (Dougherty et al., 2010).

Thereafter, there was evidence of a continuing and progressively amplified immunological and inflammatory response. There was predicted upregulation of inflammatory mediators such as TNFα and macrophage migration inhibitory factor (MIF), and increases in a variety of inflammation-associated MAP kinases, as well as enhanced expression of periostin, which is produced by epithelial cells and fibroblasts in response to IL-4 and IL-13 signalling associated with Th2-driven inflammation (Takayama et al., 2006; Corren et al., 2011). At day 77 there was also predicted upregulation of the CD69 and CD28 antigens, both of which play roles in T-cell activation in allergic inflammation (Kimzey et al., 2004; Miki-Hosokawa et al., 2009).

In parallel, based on analysis of altered miRNAs in the airway wall, several mediators and receptors associated with airway wall remodelling were also predicted to be upregulated. Among these, IGF-1 was consistently identified as a relevant growth factor from as early as day 49, with other growth factors such as FGF-1 being expressed later, together with increased expression of receptors for PDGF and TGF-β, and upregulation of various growth factor signalling pathways.

However, confirmation of upregulated expression of mRNAs that were predicted targets of downregulated miRNAs was difficult, at least in part because, as inflammation develops in the airway walls, the ongoing cellular recruitment dilutes the relative expression of mRNA by structural cells and increases the hypoxanthine-guanine phosphoribosyl transferase (HPRT) denominator relative to which mRNA expression is normalised. We have previously shown that, in this setting, it is possible for substantially increased expression of growth factors by a minority of cells, such as airway epithelial cells, to be completely masked and indeed to apparently decrease (Herbert et al., 2008). Despite this, we were able to demonstrate significantly increased expression of *Igf1* in airway wall tissue in this model. However, the dilution effect could have accounted for the apparent progressive decline in relative expression of *Tgfbr1* that we observed over time.

The evidence of increased expression of *Igf1* is consistent with our earlier published evidence in a related animal model of chronic asthma, in which upregulation of *Igf1*, *Fgf1* and *Tgfb1* mRNA was demonstrated in the airway epithelium (Herbert et al., 2008). The data are also consistent with our published evidence of significant airway remodelling, including epithelial thickening and subepithelial fibrosis, in this animal model of childhood asthma (Siegle et al., 2010).

It was of interest that there seemed to be a reciprocal relationship between the predicted expression of the type 1 and type 2 receptors for bone morphogenetic proteins, as well as for the type 2a and 2b receptors for activin. Both of these growth factors are linked to airway remodelling in human asthmatics (Karagiannidis et al., 2006; Pegorier et al., 2010), although the biological significance of this predicted pattern of regulation of receptor expression is unclear. Other intriguing predicted targets included several homeobox genes and a small number of transcription factors, notably *Hmgb3* and *Pparg*, which might be of interest in the context of the reported regulatory role of peroxisome proliferator-activated receptor-γ (PPARγ) in airway inflammation and remodelling (Lee et al., 2006).

Assessment of pulmonary CD4^+^ T cells revealed additional findings that were of considerable interest in the context of epigenetic regulation of T-cell commitment. Notably, there was early and significant demethylation of CpG sites in the promoter region of the *Il4* gene. In addition, demethylation of promoter sites in *Ifng* was demonstrated at the end of the period of challenge. Although the change in percentage demethylation was not large, it could be biologically significant, because a 5% decrease in methylation of the *Ifng* promoter has been shown to be associated with an almost threefold increase in the expression of *Ifng* mRNA (Gonsky et al., 2009). These data on epigenetic regulation of the commitment of CD4^+^ T cells to an allergic immunological response are concordant with our earlier studies demonstrating that, in related models of asthma in adult animals, IFN-γ plays a significant role in the pathogenesis (Kumar et al., 2004; Kumar et al., 2012). They might also be of relevance to childhood asthma, because birth cohort studies indicate that non-Th2 cytokines, especially IFN-γ, modify the risk of development of childhood asthma in atopic subjects and contribute to the progression of disease (Heaton et al., 2005; Hollams et al., 2009). However, in previous studies we have not found evidence of enhanced expression of IFN-γ by pulmonary CD4^+^ T cells in the model used for these studies (Siegle et al., 2010), suggesting that demethylation at *Ifng* promoter sites at this time point was indicative of potential rather than actual expression of this gene.

In conclusion, our results indicate that chronic challenge with allergen in this model of childhood asthma is associated with altered miRNA regulation, leading to predicted upregulation of genes associated with a Th2 pattern of immunological response, as well as various other pro-inflammatory mediators. This is paralleled by upregulation of genes for growth factors associated with airway wall remodelling. Collectively, these might constitute a biologically relevant pathway of gene activation associated with the development of asthma. Importantly, disease progression is also associated with altered DNA methylation that promotes stable differentiation of pulmonary CD4^+^ T cells towards a Th2 phenotype.

## MATERIALS AND METHODS

### Animals

Specific pathogen-free female BALB/c mice (either timed pregnant or aged ∼8 weeks) were obtained from Animal Services, University of Newcastle, Australia or from the Biological Resources Centre, University of New South Wales, Australia. Animals were held in individually ventilated cages, exposed to a 12-hour light-dark cycle and provided autoclaved food and water *ad libitum*. All experimental procedures complied with the requirements of the Animal Care and Ethics Committee of the University of New South Wales, Australia (ref. nos 06/119B and 09/124A).

### PVM infection and allergen sensitisation

Early-life viral infection and allergen sensitisation was performed at the University of Newcastle, Australia as previously described (Siegle et al., 2010). Infection was with mouse passaged PVM J3666 strain [∼1×10^5^ plaque-forming units (Pfu)/ml]. On days 1 and 2 of life, mice from appropriate groups were intranasally inoculated with 2 PFU in 5 μl phosphate buffered saline (PBS) on the external nares. Intranasal sensitisation to chicken egg OVA (Grade V, ≥98% pure, Sigma Australia, Australia) was performed at days 1 and 2 of life or at days 28 and 29, with 5 μg OVA/5 μl PBS and 100 μg OVA/40 μl, respectively.

### Inhalational challenge

Inhalational challenge with aerosolised OVA was performed as previously described (Siegle et al., 2006). Briefly, commencing at 7 weeks of age, BALB/c mice were exposed to ∼3 mg OVA/m^3^ for 30 minutes/day, 3 days/week for 4 weeks. This was followed by a single challenge of ∼30 mg/m^3^ for 30 minutes to induce the changes of acute inflammation. During inhalation exposures, mice were held in flow-through wire cage racks (Unifab Corporation, Kalamazoo, MI). Filtered air was drawn through the inhalation chamber (0.5 m^3^) at a flow rate of 250 l/minute and an aerosol of OVA was generated by controlled delivery of compressed air to a sidestream nebuliser (Trimed, Australia). Particle concentration within the chamber was continuously monitored using a DustTrak 8520 instrument (TSI, MN).

### Experimental groups

**Fig. 5.**
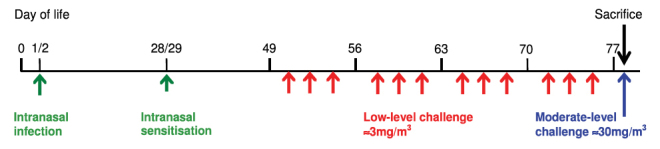
**Timeline of infection with PVM, respiratory sensitisation and inhalational challenges in the early-life model.**

In animals that had been infected with PVM at days 1 and 2 of life and intranasally sensitised to OVA at days 28 and 29 of life, miRNA expression in the airway wall was assessed at day 49 (before inhalational challenge), day 56, day 63 and day 77 (after completion of inhalational challenge) (Fig. 5). DNA methylation in CD4^+^ T cells was compared at day 49 and day 77. Experimental groups comprised six to eight animals. For miRNA studies, tissue from pairs of animals was pooled to yield four samples per group. Mice were killed by exsanguination following an overdose of sodium pentobarbital, at 4 hours after the final inhalational challenge. Controls were untreated naive mice.

### Isolation of proximal airway tissue

Airway tissue was isolated by blunt dissection (Herbert et al., 2008) using two pairs of forceps to separate lung parenchyma from the larger airways and leaving several generations of airway attached to the trachea. Airway tissue was frozen in liquid nitrogen and stored at −80°C until RNA extraction was performed.

### Isolation of mRNA and miRNA

For assessment of miRNA, total RNA was isolated from blunt dissected distal airway tissue using the mirVana miRNA isolation kit (Ambion) (Collison et al., 2011). For assessment of mRNA, RNA was isolated using TriReagent (Sigma) and, following DNase treatment (Turbo DNase, Ambion), samples were reverse transcribed into cDNA using Superscript III (Invitrogen).

### miRNA microarray

Total RNA (100 ng) was dephosphorylated and ligated with pCp-Cy3 using the Agilent miRNA labelling reagent. Labelled RNA was purified and hybridised to Agilent mouse miRNA arrays V2 with probes for Sanger miRBase version 12. Images were scanned on a G2505B Microarray Scanner (Agilent Technologies), gridded and analysed using Agilent feature extraction software version 9.5.3. Analysis of microarray data was conducted using Genespring GX 11 software (Agilent). Percentile shift normalisation (75th percentile) was performed with subsequent fold-change calculations conducted against mean normalised naive expression levels.

### Isolation of pulmonary CD4^+^ T cells

After perfusion with saline to remove blood from the pulmonary capillary bed, lungs from three animals were pooled and diced into fine fragments. Tissue was disaggregated using a mixture of type IV collagenase and DNase as previously described (Herbert et al., 2010). Lymph nodes surrounding the trachea and main bronchi were collected from pairs of animals and mechanically disaggregated. CD4^+^ T cells were isolated from the recovered cells using a FlowComp™ Mouse CD4 magnetic bead isolation kit (Invitrogen, Australia) according to the manufacturer’s instructions.

### qRT-PCR

qRT-PCR for miRNA was performed using TaqMan Expression Assays for the respective miRNA (Applied Biosystems) (Collison et al., 2011). miRNA expression was normalised to *sno202RNA*. qRT-PCR for mRNA expression used primers that were custom-designed in house. Reactions were performed using a Mastercycler-ep Realplex (Eppendorf). Amplified products were detected using SYBR green and expression was normalised to HPRT.

### CpG pyrosequencing

DNA was extracted from 1×10^6^ CD4^+^ T cells using QuickExtract solution (Epicentre Biotechnologies, Madison, WI). Extracted DNA was bisulphite converted using the EZ DNA Methylation-Gold™ kit (Zymo Research) according to the manufacturer’s instructions. Briefly, CT conversion reagent was added to each sample and bisulphite conversion was carried out in a GeneAmp PCR System 9700 thermocycler (Perkin Elmer, MA) at 98°C for 10 minutes and 64°C for 4 hours. Subsequently, bisulphite-converted DNA was desulphonated and stored at −80°C until further use.

To assess DNA methylation levels, CpG pyrosequencing was performed on the bisulphite-treated DNA. Selection of CpG sites for evaluation was based on published sources (Jones and Chen, 2006; Liu et al., 2008) or manual identification of CpG dinucleotides. Biotin-labelled primers were designed using Primer3 software online, using a published optimised approach (Shen et al., 2007). Nested pyrosequencing primers of 18–25 bases were designed within each PCR product complementary to the biotinylated template strand. Pyrosequencing was performed across the designated CpG sites on the PyroMark ID instrument using PyroGold reagents, and the relative levels of methylation at each CpG site were measured using PyroMark CpG software (Biotage).

### Statistical analysis

qRT-PCR and DNA methylation data were analysed by a oneway ANOVA followed by a Holm-Sidak multiple comparison test. The software package GraphPad Prism 6.02 (GraphPad Software, San Diego, CA) was used for data analysis and preparation of graphs.

## References

[b1-0060993] Collison A. Herbert C. Siegle J. S. Mattes J. Foster P. S. Kumar R. K. (2011). Altered expression of microRNA in the airway wall in chronic asthma: miR-126 as a potential therapeutic target. BMC Pulm. Med. 11, 29 21605405 10.1186/1471-2466-11-29 PMC3116478

[b2-0060993] Corren J. Lemanske R. F. Hanania N. A. Korenblat P. E. Parsey M. V. Arron J. R. Harris J. M. Scheerens H. Wu L. C. Su Z. (2011). Lebrikizumab treatment in adults with asthma. N. Engl. J. Med. 365, 1088–1098 21812663 10.1056/NEJMoa1106469

[b3-0060993] Domachowske J. B. Bonville C. A. Rosenberg H. F. (2004). Animal models for studying respiratory syncytial virus infection and its long term effects on lung function. Pediatr. Infect. Dis. J. 23 Suppl, S228–S234 15577578 10.1097/01.inf.0000144672.81955.a4

[b4-0060993] Dougherty R. H. Sidhu S. S. Raman K. Solon M. Solberg O. D. Caughey G. H. Woodruff P. G. Fahy J. V. (2010). Accumulation of intraepithelial mast cells with a unique protease phenotype in T(H)2-high asthma. J. Allergy Clin. Immunol. 125, 1046–1053 e1048 20451039 10.1016/j.jaci.2010.03.003 PMC2918406

[b5-0060993] Gonsky R. Deem R. L. Targan S. R. (2009). Distinct methylation of IFNG in the gut. J. Interferon Cytokine Res. 29, 407–414 19450149 10.1089/jir.2008.0109 PMC2956574

[b6-0060993] Heaton T. Rowe J. Turner S. Aalberse R. C. de Klerk N. Suriyaarachchi D. Serralha M. Holt B. J. Hollams E. Yerkovich S. (2005). An immunoepidemiological approach to asthma: identification of in-vitro T-cell response patterns associated with different wheezing phenotypes in children. Lancet 365, 142–149 15639296 10.1016/S0140-6736(05)17704-6

[b7-0060993] Herbert C. Hettiaratchi A. Webb D. C. Thomas P. S. Foster P. S. Kumar R. K. (2008). Suppression of cytokine expression by roflumilast and dexamethasone in a model of chronic asthma. Clin. Exp. Allergy 38, 847–856 18307529 10.1111/j.1365-2222.2008.02950.x

[b8-0060993] Herbert C. Scott M. M. Scruton K. H. Keogh R. P. Yuan K. C. Hsu K. Siegle J. S. Tedla N. Foster P. S. Kumar R. K. (2010). Alveolar macrophages stimulate enhanced cytokine production by pulmonary CD4+ T-lymphocytes in an exacerbation of murine chronic asthma. Am. J. Pathol. 177, 1657–1664 20724599 10.2353/ajpath.2010.100019 PMC2947263

[b9-0060993] Hollams E. M. Deverell M. Serralha M. Suriyaarachchi D. Parsons F. Zhang G. de Klerk N. Holt B. J. Ladyman C. Sadowska A. (2009). Elucidation of asthma phenotypes in atopic teenagers through parallel immunophenotypic and clinical profiling. J. Allergy Clin. Immunol. 124, 463–470, e1–e16 19733295 10.1016/j.jaci.2009.06.019

[b10-0060993] Holt P. G. Sly P. D. (2009). Non-atopic intrinsic asthma and the ‘family tree’ of chronic respiratory disease syndromes. Clin. Exp. Allergy 39, 807–811 19400902 10.1111/j.1365-2222.2009.03258.x

[b11-0060993] Holt P. G. Sly P. D. (2012). Viral infections and atopy in asthma pathogenesis: new rationales for asthma prevention and treatment. Nat. Med. 18, 726–735 22561836 10.1038/nm.2768

[b12-0060993] Holt P. G. Strickland D. H. (2010). Interactions between innate and adaptive immunity in asthma pathogenesis: new perspectives from studies on acute exacerbations. J. Allergy Clin. Immunol. 125, 963–972, quiz 973–974. 20394979 10.1016/j.jaci.2010.02.011

[b13-0060993] Jackson D. J. Evans M. D. Gangnon R. E. Tisler C. J. Pappas T. E. Lee W. M. Gern J. E. Lemanske R. F. Jr (2012). Evidence for a causal relationship between allergic sensitization and rhinovirus wheezing in early life. Am. J. Respir. Crit. Care Med. 185, 281–285 21960534 10.1164/rccm.201104-0660OC PMC3297109

[b14-0060993] Jardim M. J. Dailey L. Silbajoris R. Diaz-Sanchez D. (2012). Distinct microRNA expression in human airway cells of asthmatic donors identifies a novel asthma-associated gene. Am. J. Respir. Cell Mol. Biol. 47, 536–542 22679274 10.1165/rcmb.2011-0160OC

[b15-0060993] Jones B. Chen J. (2006). Inhibition of IFN-gamma transcription by site-specific methylation during T helper cell development. EMBO J. 25, 2443–2452 16724115 10.1038/sj.emboj.7601148 PMC1478170

[b16-0060993] Karagiannidis C. Hense G. Martin C. Epstein M. Rückert B. Mantel P. Y. Menz G. Uhlig S. Blaser K. Schmidt-Weber C. B. (2006). Activin A is an acute allergen-responsive cytokine and provides a link to TGF-beta-mediated airway remodeling in asthma. J. Allergy Clin. Immunol. 117, 111–118 16387593 10.1016/j.jaci.2005.09.017

[b17-0060993] Kimzey S. L. Liu P. Green J. M. (2004). Requirement for CD28 in the effector phase of allergic airway inflammation. J. Immunol. 173, 632–640 15210826 10.4049/jimmunol.173.1.632

[b18-0060993] Kumar R. K. Herbert C. Webb D. C. Li L. Foster P. S. (2004). Effects of anticytokine therapy in a mouse model of chronic asthma. Am. J. Respir. Crit. Care Med. 170, 1043–1048 15306533 10.1164/rccm.200405-681OC

[b19-0060993] Kumar R. K. Hitchins M. P. Foster P. S. (2009). Epigenetic changes in childhood asthma. Dis. Model. Mech. 2, 549–553 19892885 10.1242/dmm.001719

[b20-0060993] Kumar R. K. Siegle J. S. Kaiko G. E. Herbert C. Mattes J. E. Foster P. S. (2011). Responses of airway epithelium to environmental injury: role in the induction phase of childhood asthma. J. Allergy (Cairo) 2011, 257017 22574070 10.1155/2011/257017 PMC3206385

[b21-0060993] Kumar R. K. Yang M. Herbert C. Foster P. S. (2012). Interferon-γ, pulmonary macrophages and airway responsiveness in asthma. Inflamm. Allergy Drug Targets 11, 292–297 22856520 10.2174/187152812800958951

[b22-0060993] Kusel M. M. de Klerk N. H. Kebadze T. Vohma V. Holt P. G. Johnston S. L. Sly P. D. (2007). Early-life respiratory viral infections, atopic sensitization, and risk of subsequent development of persistent asthma. J. Allergy Clin. Immunol. 119, 1105–1110 17353039 10.1016/j.jaci.2006.12.669 PMC7125611

[b23-0060993] Kusel M. M. Kebadze T. Johnston S. L. Holt P. G. Sly P. D. (2012). Febrile respiratory illnesses in infancy and atopy are risk factors for persistent asthma and wheeze. Eur. Respir. J. 39, 876–882 21920891 10.1183/09031936.00193310

[b24-0060993] Lee K. S. Park S. J. Kim S. R. Min K. H. Jin S. M. Lee H. K. Lee Y. C. (2006). Modulation of airway remodeling and airway inflammation by peroxisome proliferator-activated receptor gamma in a murine model of toluene diisocyanate-induced asthma. J. Immunol. 177, 5248–5257 17015710 10.4049/jimmunol.177.8.5248

[b25-0060993] Liu J. Ballaney M. Al-alem U. Quan C. Jin X. Perera F. Chen L. C. Miller R. L. (2008). Combined inhaled diesel exhaust particles and allergen exposure alter methylation of T helper genes and IgE production in vivo. Toxicol. Sci. 102, 76–81 18042818 10.1093/toxsci/kfm290 PMC2268643

[b26-0060993] Martino D. Prescott S. (2011). Epigenetics and prenatal influences on asthma and allergic airways disease. Chest 139, 640–647 21362650 10.1378/chest.10-1800

[b27-0060993] Miki-Hosokawa T. Hasegawa A. Iwamura C. Shinoda K. Tofukuji S. Watanabe Y. Hosokawa H. Motohashi S. Hashimoto K. Shirai M. (2009). CD69 controls the pathogenesis of allergic airway inflammation. J. Immunol. 183, 8203–8215 19923457 10.4049/jimmunol.0900646

[b28-0060993] Pegorier S. Campbell G. A. Kay A. B. Lloyd C. M. (2010). Bone morphogenetic protein (BMP)-4 and BMP-7 regulate differentially transforming growth factor (TGF)-beta1 in normal human lung fibroblasts (NHLF). Respir. Res. 11, 85 20573231 10.1186/1465-9921-11-85 PMC2898775

[b29-0060993] Rosenberg H. F. Domachowske J. B. (2008). Pneumonia virus of mice: severe respiratory infection in a natural host. Immunol. Lett. 118, 6–12 18471897 10.1016/j.imlet.2008.03.013 PMC2494858

[b30-0060993] Sabroe I. Parker L. C. Dockrell D. H. Davies D. E. Dower S. K. Whyte M. K. (2007). Targeting the networks that underpin contiguous immunity in asthma and chronic obstructive pulmonary disease. Am. J. Respir. Crit. Care Med. 175, 306–311 17138954 10.1164/rccm.200606-777PP

[b31-0060993] Sel S. Wegmann M. Dicke T. Sel S. Henke W. Yildirim A. O. Renz H. Garn H. (2008). Effective prevention and therapy of experimental allergic asthma using a GATA-3-specific DNAzyme. J. Allergy Clin. Immunol. 121, 910–916, e5 18325571 10.1016/j.jaci.2007.12.1175

[b32-0060993] Shen L. Guo Y. Chen X. Ahmed S. Issa J. P. (2007). Optimizing annealing temperature overcomes bias in bisulfite PCR methylation analysis. Biotechniques 42, 48–58, 50, 52 passim. 17269485 10.2144/000112312

[b33-0060993] Siegle J. S. Hansbro N. Herbert C. Yang M. Foster P. S. Kumar R. K. (2006). Airway hyperreactivity in exacerbation of chronic asthma is independent of eosinophilic inflammation. Am. J. Respir. Cell Mol. Biol. 35, 565–570 16794258 10.1165/rcmb.2006-0135OC

[b34-0060993] Siegle J. S. Hansbro N. Herbert C. Rosenberg H. F. Domachowske J. B. Asquith K. L. Foster P. S. Kumar R. K. (2010). Early-life viral infection and allergen exposure interact to induce an asthmatic phenotype in mice. Respir. Res. 11, 14 20122285 10.1186/1465-9921-11-14 PMC2842242

[b35-0060993] Sly P. D. Boner A. L. Björksten B. Bush A. Custovic A. Eigenmann P. A. Gern J. E. Gerritsen J. Hamelmann E. Helms P. J. (2008). Early identification of atopy in the prediction of persistent asthma in children. Lancet 372, 1100–1106 18805338 10.1016/S0140-6736(08)61451-8 PMC4440493

[b36-0060993] Sly P. D. Kusel M. Holt P. G. (2010). Do early-life viral infections cause asthma? J. Allergy Clin. Immunol. 125, 1202–1205 20304476 10.1016/j.jaci.2010.01.024

[b37-0060993] Takayama G. Arima K. Kanaji T. Toda S. Tanaka H. Shoji S. McKenzie A. N. Nagai H. Hotokebuchi T. Izuhara K. (2006). Periostin: a novel component of subepithelial fibrosis of bronchial asthma downstream of IL-4 and IL-13 signals. J. Allergy Clin. Immunol. 118, 98–104 16815144 10.1016/j.jaci.2006.02.046

[b38-0060993] Taussig L. M. Wright A. L. Holberg C. J. Halonen M. Morgan W. J. Martinez F. D. (2003). Tucson Children’s Respiratory Study: 1980 to present. J. Allergy Clin. Immunol. 111, 661–675, quiz 676. 12704342 10.1067/mai.2003.162

[b39-0060993] van Panhuys N. Le Gros G. McConnell M. J. (2008). Epigenetic regulation of Th2 cytokine expression in atopic diseases. Tissue Antigens 72, 91–97 18554247 10.1111/j.1399-0039.2008.01068.x

[b40-0060993] Zhu J. Paul W. E. (2008). CD4 T cells: fates, functions, and faults. Blood 112, 1557–1569 18725574 10.1182/blood-2008-05-078154 PMC2518872

